# Post-treatment Lyme disease syndrome symptomatology and the impact on life functioning: is there something here?

**DOI:** 10.1007/s11136-012-0126-6

**Published:** 2012-02-01

**Authors:** John N. Aucott, Alison W. Rebman, Lauren A. Crowder, Kathleen B. Kortte

**Affiliations:** 1 Department of Medicine, Johns Hopkins University School of Medicine, 10755 Falls Road, Suite 200, Lutherville, MD 21093 USA; 2 The Lyme Disease Research Foundation of Maryland, 10755 Falls Road, Suite 200, Lutherville, MD 21093 USA; 3 Department of Physical Medicine and Rehabilitation, Johns Hopkins University School of Medicine, Phipps 174, 600 North Wolfe Street, Baltimore, MD 21287 USA

**Keywords:** Post-treatment Lyme disease syndrome, Chronic disease, Depression, Life functioning, Outcomes

## Abstract

**Purpose:**

A subset of patients treated for Lyme disease report persistent or recurrent symptoms of unknown etiology named post-treatment Lyme disease syndrome (PTLDS). This study aims to describe a cohort of participants with early, untreated Lyme disease, and characterize post-treatment symptomatology and functional impact of PTLDS over time.

**Methods:**

Sixty-three participants with erythema migrans and systemic symptoms were enrolled in a prospective cohort study. Participants underwent physical exams and clinical assessments, and completed the SF-36 (daily life functioning) and the Beck Depression Inventory, Second Edition (BDI-II) (depression), at each of five visits over a period of 6 months.

**Results:**

Signs of Lyme disease disappeared post-treatment; however, new-onset patient-reported symptoms increased or plateaued over time. At 6 months, 36% of patients reported new-onset fatigue, 20% widespread pain, and 45% neurocognitive difficulties. However, less than 10% reported greater than “minimal” depression across the entire period. Those with PTLDS (36%) did not differ significantly from those without with respect to demographics, pre-treatment SF-36, and BDI-II scores. Statistically significant differences were found over time on the Role Physical, Vitality, Social Functioning, Role Emotional, and Mental Health subscales (with a trend toward significance for the remaining three subscales of Physical Functioning, Bodily Pain, and General Health) of the SF-36 between those with an eventual PTLDS diagnosis and those without when measured at 6 months.

**Conclusions:**

Unlike clinical signs of Lyme disease, new-onset symptoms are reported by a subset of participants without evidence of depressive symptomatology. Patients who developed PTLDS had significantly lower life functioning compared to those without PTLDS. We propose future avenues for researching infection-triggered symptoms resulting from multiple mechanisms.

## Introduction

Lyme disease, caused by the spirochete bacteria *Borrelia burgdorferi*, is the most common vector-borne infectious disease in North America. More than 38,000 new cases were reported in the United States in 2009 [1], but underreporting is estimated to be 6- to 12-fold, making the true number likely over 100,000 cases per year [2]. Clinical findings in early Lyme disease range from erythema migrans (EM) rash with or without “viral-like” systemic symptoms to patients presenting with symptoms in the absence of a diagnostic EM rash [3, 4]. The infection may cause either localized or disseminated disease, with sensitive measures showing rates of blood borne infection as high as 70% [5]. Early disseminated infection may be associated with VII nerve palsy, cardiac disease, meningitis, and rarely, evidence of encephalitis [6]. When untreated, 60% of cases may develop “late” Lyme disease with joint pain and arthritis [6]. Less common features of late disease include neuropathy and chronic encephalopathy manifesting as memory deficits, concentration difficulties, and fatigue. However, encephalitis with focal abnormalities on neuroimaging is rare in the United States [7]. Patient-reported symptoms, such as fatigue, cognitive dysfunction, and musculoskeletal pain, are common in both early and late phases of untreated illness [8, 9].

Erythema migrans rash and other early disease signs respond to antibiotic treatment, which also largely prevents later objective manifestations of disease [10]. However, 40–50% of patients in early treatment studies reported persistent or recurrent symptoms including headache, musculoskeletal pain, and lethargy [10, 11]. More recent trials in ideally treated patients show improved outcomes, but continue to document persistent or recurring symptoms in as many as 17% of patients up to 12 months after treatment [12].

Over time, a pattern of findings emerged in the literature supporting the persistence of symptoms in a subgroup of individuals who had received treatment [13, 14]. The term post-treatment Lyme disease syndrome (PTLDS) was coined to capture the pattern symptoms when they persist for longer than 6 months post-treatment [15]. The Infectious Disease Society of America (IDSA) soon followed with a case definition of PTLDS that includes a documented episode of early or late Lyme disease with post-treatment resolution of objective symptoms of Lyme disease, but subsequent onset of symptoms of fatigue, widespread musculoskeletal pain, and/or complaints of cognitive difficulties. These subjective symptoms must be continuous or relapsing for at least 6 months following completion of treatment and must be severe enough to reduce functional ability in the patient’s life [15]. Retrospective studies of the long-term implications of PTLDS have shown that these symptoms may persist for years [16, 17] and negatively impact global life and Physical Functioning [18, 19].

As suggested by Sigal and Hassett [20] over 5 years ago, there is a need for a prospective study of individuals with proven Lyme disease who are tracked over time to capture the development and course of symptoms leading to PTLDS. To date, no prospective cohort studies of early Lyme disease in North America have been published to examine the frequency, severity, and impact on life functioning of patients who develop PTLDS versus those that do not develop PTLDS. The aim of the current study is to address this gap in the literature. As such, in the current study, a low-risk patient sample with systemic signs and symptoms of Lyme disease, but no other recognized risk factors of PTLDS, were tracked over a 6-month period of time after diagnosis and treatment. We hypothesize that those patients who develop PTLDS will have a more negative impact of their health status on life functioning over time as compared with patients whose symptoms resolve after treatment.

## Methods

The current study is part of a larger, ongoing prospective cohort study of consecutive patients with Lyme disease being conducted in a suburban community of a medium-sized, Mid-Atlantic city since the summer of 2008. Adult patients from a healthy, ambulatory population were identified during clinical evaluations of skin lesions or flu-like or “viral-like” symptoms in the urgent care facility or by one of 20 primary care practitioners at a suburban medical facility. Patients were referred to a primary care physician (JNA) who has infectious disease training and were invited to participate if the clinical diagnosis of EM was confirmed. The study was approved by the Johns Hopkins Medicine Institutional Review Board.

Eligible participants are required to be treatment-naïve and to have evidence of systemic disease; typically manifesting as dissemination of the primary EM lesion or concurrent onset of new viral-like or other symptoms. Patients with a prior history of Lyme disease are excluded. Patients’ self-reporting pre-existing conditions including chronic fatigue syndrome, fibromyalgia, major depressive disorders, cancer, or autoimmune conditions were excluded. Exclusion criteria were based on the proposed IDSA [15] case definition of PTLDS, in order to minimize the impact of medical comorbidities linked to our outcome variables of fatigue, pain, and cognitive dysfunction.

### Study design and timeline

After consenting to participate, study participants completed an initial visit during their acute illness and then were followed over a 6-month period of time, including visits occurring after completion of a three-week course of doxycycline, at 4 weeks post-treatment, 3 months post-treatment, and 6 months post-treatment. During the initial, pre-treatment study visit, self-reported demographic and medical history data and two-tier antibody testing for *B. burgdorferi* were performed. At all study visits, participants underwent a physical exam, were asked about self-reported symptom that were present during the prior interval, and completed self-administered, standardized surveys.

### Symptom reporting

Patients’ self-reported symptoms were elicited at all visits through a structured interview using a standardized written questionnaire of 37 symptoms. Since a validated symptom checklist does not exist for Lyme disease, this questionnaire was developed through a review of the literature and interviews with patients with a history of Lyme disease. At the initial, pre-treatment study visit, participants reported the presence of symptoms that included items, such as fatigue, musculoskeletal pain, neurocognitive difficulties, fever, chills, or sleep disturbance observed concurrently with their acute illness. At subsequent study visits, participants reported the presence of any new onset of these symptoms that had occurred during the previous follow-up period and that had not, in their estimation, pre-dated their acute Lyme disease. Participants were instructed to report symptoms as absent, improved, same, worse, new, or returned since the previous study visit. Interviewers administered the questionnaire in a consistent fashion and did not probe for specific symptoms.

As post-infectious symptoms have been described as waxing and waning over time, we did not require symptoms to be present at the day of the study visit, only that they were experienced during the prior interval. Following IDSA case definition, participants were considered to have PTLDS if they reported the presence of new-onset fatigue, widespread musculoskeletal pain, or neurocognitive difficulties at their 6-month study visit. Fatigue was defined as self-report of new or worsened fatigue since diagnosis. Widespread musculoskeletal pain was defined as the presence of muscle or joint pain in more than one region of the body. Neurocognitive symptoms were defined as the self-reported presence of trouble focusing or concentrating, difficulty with word-finding, or difficulty remembering information.

### Depression

Given that depression symptomatology has been hypothesized to play a role in the development of PTLDS, the Beck Depression Inventory, Second Edition (BDI-II) [21], was administered at each visit. The BDI-II has been validated in a variety of samples including both non-clinical [22] and clinically depressed adults [23]. Each of the 21 items in the scale is rated from 0 to 3; thus, the total score represents a range from 0 to 63, with cutoffs of 0–13 (“minimal” depression), 14–19 (“mild” depression), 20–28 (“moderate” depression), and 29–63 (“severe” depression) [21]. Internal consistency was found to be acceptable in our sample (α = 0.86).

### Impact on life functioning

In order to capture the impact of health status on life functioning, the 36-item Short Form Health Survey, Version 2 (SF-36), was administered at each study visit. It has been designed to study eight health attributes and has been shown to have high reliability and validity across a range of populations [24]. Each of the 36 items in the measure loads onto one of eight subscales: Physical Functioning, Role Physical, Bodily Pain, General Health, Vitality, Social Functioning, Role Emotional, and Mental Health. Raw scores of 0–100 are generated for each subscale, and then scores are adjusted using a linear transformation to a mean of 50 and a standard deviation of 10 using 1998 general population norms [24]. Lower scores reflect more negative impact of health status on life functioning. In our sample, we found Cronbach’s α of >0.70 for all subscales, with 5/8 subscales >0.90.

### Statistical analyses

Sample characteristics and temporal trends are characterized using simple descriptive statistics. Cross-sectional differences by PTLDS status were tested using Chi-square and independent sample *t* tests for demographic variables. Each of the eight SF-36 subscale scores for those with PTLDS (PTLDS-positive) was compared with those without PTLDS (PTLDS-negative) over time using separate linear regression models with generalized estimating equations to account for the statistical dependence incurred by repeated measures of each outcome on the same individual [25]. Given the small sample size and to reduce the type 1 error rate, a more conservative alpha level of *p* <0.01 was considered statistically significant for all tests. Data were analyzed using SAS (Statistical Analysis System Institute, Cary, NC, USA).

## Results

### Cohort characteristics

Sixty-five patients with early Lyme disease were enrolled in the study at the time of analysis. Two participants whose initial BDI-II scores indicated the possibility of undiagnosed moderate-to-severe depression at study entry were subsequently removed, thus a total of 63 participants were included in the analysis. The demographic and baseline medical characteristics of the sample are shown in Table 1. This participant sample was highly active and healthy prior to the onset of Lyme disease. Participants reported the presence of an average of one medical diagnosis, such as hypertension, thyroid disease, and hyperlipidemia, that are not typically associated with limitations in health function. Participants were on an average of one prescription medication, and participant demographics showed a highly educated sample who are within a high-income bracket. Distributions of race, sex, and education are similar to those previously reported for Lyme disease [18]. For all measured variables, the response rate was 97.1% (306/315) for all five measured time points, with a response rate of 95.2% (60/63) for the 6-month follow-up visit.

**Table 1:** Baseline demographic characteristics of early Lyme cohort (*n* = 63)

Characteristic	Mean ± SD (range)
Age (years)	48.9 ± 15.5 (20–75)
Formal schooling (years)	16.12 ± 2.37 (11–21)
Income (dollars)	139,833 ± 110,871 (27,000–500,000)^a^
Number of additional diagnoses	1.33 ± 0.53 (0–5)
Number of prescriptions	1.0 ± 1.59 (0–6)

	*N* (%)
Sex	
Male	35 (56)
Female	28 (44)
Race	
White, non-Hispanic	61 (95)
White, Hispanic	1 (2)
Other, non-Hispanic	1 (2)
Education	
Some high school	1 (2)
High school graduate	6 (9)
Some college	13 (21)
College graduate	20 (32)
Graduate/professional	23 (36)

^a^Six patients missing income data (*n* = 57)

Initial physician-observed signs are shown in Table 2. Approximately one-third of the sample presented with disseminated cutaneous EM on skin exam, and a similar proportion of the sample had at least one elevated liver function tests. Forty percent tested positive on commercial two-tier testing at their initial, pre-treatment visit; repeat testing 3 weeks later revealed an additional 27% had seroconverted during the treatment interval. Six participants (10%) were subsequently retreated for primary treatment failures, including three with new neurologic abnormalities documented on nerve conduction studies and three whose primary EM rash enlarged during antibiotic treatment.

**Table 2:** Initial physical exam and laboratory findings of early Lyme cohort (*n* = 63)

Characteristic	Mean ± SD (range)	*N* (%)
Erythema migrans rash		
Size of primary rash (cm^2^)	133.1 ± 125.1 (15–594)	
Single		43 (68)
Disseminated		20 (32)
Physical exam abnormalities		
Fever ≥ 38.0°C^a^		3 (5)
Lymphadenopathy		9 (14)
Liver span		5 (8)
Spleen tip		2 (3)
Illness duration (days)	7.9 ± 6.2 (1–35)	
Seropositive based on two-tier algorithm		25 (40)
Lymphocytes		
Absolute count, 10^3^ (μL)	1.3 ± 0.6 (0.3–3.4)	
>1.10 × 10^3^ (μL)		24 (38)
Liver function tests^b^		
AST (U/L)	43.7 ± 62.1 (10–413)	
ALT (U/L)	51.5 ± 94.9 (10–704)	
AST > 35 U/L or ALT > 40 U/L		23 (37)

*AST* aspartate aminotransferase, *ALT* alanine aminotransferase

^a^One patient missing physical exam temperature reading (*n* = 62)

^b^One patient missing liver function tests (*n* = 62)

Table 3 indicates that fatigue, headache, fever, sweats, and chills were the most frequently reported symptoms of acute illness. Notably, while 60% of participants reported fever as part of their illness, it was documented at the time of physical exam for only 3%. The initial BDI-II score of the cohort fell well within the low end of the “minimal” range. Three participants in the cohort endorsed mild range of depressive symptomatology (scores 14–19) of which the majority were somatic symptoms.

**Table 3:** Initial symptoms of early Lyme cohort (*n* = 63)

Self-report on clinical exam			
≥25% of sample		<25% of sample	
Symptom	*N* (%)	Symptom	*N* (%)
Fatigue	48 (76)	Nausea	14 (22)
Headache	44 (70)	Irritability	13 (21)
Fever	38 (60)	Visual sensitivity to light	10 (16)
Sweats	38 (60)	Parasthesias	10 (16)
Chills	38 (60)	Sore throat	10 (16)
Muscle pains	34 (54)	Change in vision clarity	8 (13)
Joint pains	30 (48)	Urination changes	9 (14)
Neck pain	29 (46)	Diarrhea	7 (11)
Sleep disturbance	26 (41)	Heart palpitations	7 (11)
Dizziness	19 (30)	Tinnitus	6 (10)
Low back pain	17 (27)	Loss of coordination	6 (10)
Difficulty concentrating	15 (24)	Anxiety	6 (10)

Beck Depression Inventory II	
	Mean ± SD (range)
Total score	4.4 ± 4.3 (0–19)

Self-report symptom included in table if reported by ≥10% of the sample

### Temporal trends

**Fig. 1:**
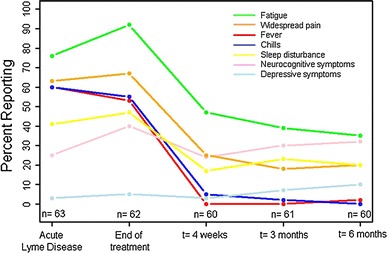
Self-reported symptoms of the cohort with acute Lyme disease over time

Figure 1 depicts self-reported symptoms at the time of diagnosis (prior to treatment) and up to 6 months following treatment. As expected, the percentage of participants reporting fever and chills, symptoms of acute illness, decreased at the first follow-up visit and returned to near 0% for all subsequent study visits. Alternatively, the percentage of participants reporting new-onset fatigue, widespread pain, and neurocognitive difficulties increased during the treatment interval and did not return to 0% after completion of treatment. The percentage of participants reporting neurocognitive difficulty was approximately 9% higher at 6 months than it was during the acute illness. Less than 10% of the sample self-reported new-onset depressive symptoms, which was mirrored in the BDI-II scores (<8% of the cohort had a score >13 at any follow-up visit).

### Group differences

**Fig. 2:**
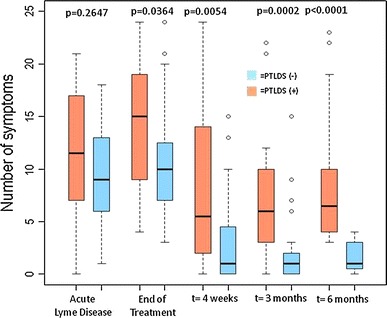
Boxplot of number of self-reported symptoms by PTLDS status over time

Using our classification of PTLDS, 35% of the sample (21 out of 63) was found to meet the case definition of PTLDS at 6 months. No statistically significant differences in demographic characteristics were found between the PTLDS-positive group (*n* = 21) and the PTLDS-negative group (*n* = 42). Final group status was then applied retrospectively. Figure 2 pictorially represents the overall number of clinically reported symptoms by PTLDS status. At the initial visit, PTLDS-positive group (mean, *M* = 11.00, standard deviation, SD = 6.26) did not report significantly more symptoms than the PTLDS-negative group (*M* = 9.23, SD = 4.48; *t* (58) = −1.27, *p* = 0.21). However, there is a statistically significant group difference in symptoms reported, which increased over time at each of the successive follow-up study visits. Similarly, PTLDS-positive group (*M* = 5.95, SD = 5.63) did not have significantly higher BDI-II scores than the PTLDS-negative group at the initial visit (*M* = 3.68, SD = 3.31; *t* (28) = −1.69, *p* = 0.10; Satterthwaite), but there was a statistically significant difference (*p* = 0.0002) after 6 months. When SF-36 scores of patients with and without PTLDS were compared, there were no differences at pre-treatment visit 1. However, scores for Physical Functioning, Role Physical, Vitality, Social Functioning, and Mental Health subscales were significantly lower in PTLDS-positive group compared with PTLDS-negative group at visit 2, immediately post-treatment (*p* = 0.0484, *p* = 0.0024, *p* = 0.0175, *p* = 0.0091, *p* = 0.0022 respectively, shown in Table 4).

**Table 4:** Norm-based scores for SF-36 measured pre- and post-treatment by PTLDS group

Instrument	PTLDS+ Pre-treatment		PTLDS− Pre-treatment		PTLDS+ Post-treatment		PTLDS− Post-treatment	
	Mean (SD)	Median	Mean (SD)	Median	Mean (SD)	Median	Mean (SD)	Median
SF-36								
Physical Functioning	47.39 (11.24)	51.77	52.03 (8.72)	55.98	49.03 (9.35)	51.77	53.70 (5.14)*	54.93
Role Physical	44.61 (9.86)	45.84	49.32 (9.42)	51.96	40.81 (10.67)	42.16	49.13 (8.87)***	51.96
Bodily Pain	45.89 (13.20)	48.18	48.86 (8.97)	50.71	48.11 (12.45)	53.25	52.87 (9.84)	55.36
General Health	52.50 (7.58)	53.65	55.13 (6.25)	55.74	51.79 (9.42)	52.93	55.22 (5.84)	55.32
Vitality	48.03 (11.55)	50.53	53.03 (10.56)	55.21	45.22 (13.23)	44.29	53.13 (10.94)*	55.21
Social Functioning	47.85 (11.09)	51.40	50.31 (9.50)	56.85	42.67 (12.18)	45.94	50.28 (9.12)**	56.85
Role Emotional	50.83 (9.19)	55.88	54.23 (4.97)	55.88	48.49 (7.87)	50.05	52.59 (8.58)	55.88
Mental Health	50.99 (9.15)	52.82	54.87 (7.44)	58.46	49.59 (6.97)	50.01	54.85 (5.40)***	55.64

Significance levels reached when comparing PTLDS+ and PTLDS− at each time point

* *p* ≤ 0.05

** *p* ≤ 0.01

*** *p* ≤ 0.0025

**Fig. 3:**
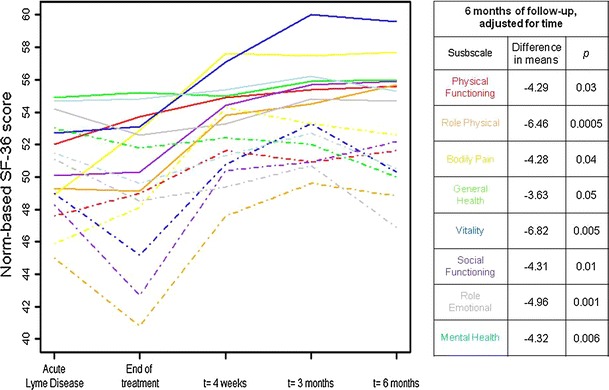
Mean SF-36 subscale scores by PTLDS group across time. *Solid lines* indicate PTLDS-negative group; *dashed lines* indicate PTLDS-positive group. A total of 60 participants with complete follow-up data up to 6 months post-treatment are included (39 PTLDS-negative and 21 PTLDS-positive at each time point). This regression was calculated using 0 for PTLDS-negative and 1 for PTLDS-positive

Figure 3 pictorially represents the pattern of norm-based SF-36 scores over the follow-up period by PTLDS status determined at the final visit. At the 6-month follow-up, the results of linear regression analyses adjusted for time revealed that the PTLDS-positive group differed significantly both statistically and in terms of minimal important change for the Role Physical and Vitality subscales (Fig. 3). All other subscales differed in terms of statistical significance, but did not meet the minimal important change criteria of greater than a five-point difference that has been determined to be clinically significant as defined in the literature for similar patient populations [26].

## Discussion

The current study provides a description of a cohort of North American patients with early Lyme disease, focusing on the course of symptomatology and impact on life functioning over a 6-month post-treatment phase. This is the first study to combine a prospective design with serial measurements of health functional outcomes, thus allowing us to characterize PTLDS and explore several proposed mechanisms for the development of PTLDS.

### Symptoms in PTLDS

Two different symptom patterns over time were found in our patients. Self-reported symptoms of acute illness (fever and chills) resolved for nearly all participants by 4 weeks post-treatment. In contrast, new-onset fatigue, widespread pain, and sleep disturbance were reported during the follow-up interval among 20–45% of participants. Neurocognitive symptoms were reported by approximately one-quarter of the cohort at the initial, pre-treatment visit and by approximately 1/3 of participants by the end of the study. Given that this is a subjective report, it may be that the impact of fatigue and pain on daily life functioning, which can serve as distractions when trying to complete life tasks, is interpreted by the patient as memory or concentration problems. Further research is needed to explore the relationship of self-reported symptoms and objective evidence of neurocognitive dysfunction (i.e., neuropsychological test data) to establish whether there is evidence of true decline in brain functioning.

Our results may differ from previous studies in important ways. Retrospective, community-based studies may include a higher proportion of patients with neurologic presentations, delayed diagnosis, and exposure to non-standard therapies. All of these known risk factors for PTLDS were limited or non-existent in our cohort. Thus, while reflective of community practice, retrospective studies may overestimate the severity of these symptoms among ideally treated patients. Conversely, our 35% PTLDS rate is somewhat higher than previous studies requiring only EM at study entry [12], or excluding patients with systemic signs or symptoms [27]. We believe that our cohort with evidence of systemic illness is representative of the majority of patients with early Lyme disease as sensitive culture-based studies show 70% of patients are blood culture–positive at the time of early diagnosis [5]. Our focus on impact of symptoms on function demonstrate that symptoms may not be as mild as previously thought with significant health-related quality of life impact and diminished function even in a previously healthy, low-risk population.

### Impact on life functioning

Our findings suggest that patients who developed PTLDS had significantly lower life functioning across the follow-up period compared with those without PTLDS. We note that differences in function are apparent and significantly different in PTLDS patients at the first post-treatment visit. This finding suggests that patients destined to develop PTLDS may be able to be identified at an early time point when they might benefit from other interventions to prevent longer-term poor functional outcomes. Our results are comparable to retrospective studies of patients with PTLDS, which have indicated that these individuals may have significantly lower functional status outcomes across most SF-36 subscales [18, 19]. It can be posited that the types of symptoms reported in PTLDS, such as pain, fatigue, sleep disruption, and neurocognitive dysfunction, would affect a range of functional domains, including role limitations resulting from physical or emotional complaints. At the final study visit, our participants with PTLDS were below the population mean in their life functioning secondary to the impact of their Physical Health, emotional distress, and Vitality. In contrast, participants who did not develop PTLDS had mean scores on all eight functional realms that were at least 0.5 SD above the population mean. Comparisons of absolute scores to the population mean are limited by the possibility of higher than average functional status among our cohort, as generated by our inclusion/exclusion criteria. We believe the demographics of our study population is similar to most other studies and patient populations concentrated in the suburban communities and resorts surrounding the major east coast metropolitan areas of the United States. Study participants like ours come from largely healthy ambulatory populations with high socioeconomic status and health status.

### Similarity to other post-treatment infectious diseases

Persistent, post-infectious symptoms of illness have also been reported following other infectious diseases. Hickie et al. [28] described disabling fatigue, musculoskeletal pain, neurocognitive difficulties, and mood disturbance in 12% of 253 participants after acute infection with Epstein-Barr virus, Q fever, or Ross River virus after 6 months. This post-infective fatigue syndrome occurred with similar incidence and presentation across the different infectious triggering events and was predicted largely by severity of the acute illness rather than by demographic, psychological, or microbiological factors. Infectious diseases, including Lyme disease, have also been implicated in post-infectious fibromyalgia, a syndrome with similar symptoms to PTLDS that has a proposed pathophysiology of “central sensitization to chronic pain” [29]. It is unknown whether these post-infectious syndromes, including those symptoms reported by our cohort, share a common mechanism that results in their similar clinical phenotypes.

### Is there something here? Possible mechanisms for the persistence of symptoms

The significance, etiology, and perpetuation of PTLDS remain poorly understood. As a result, controversy in both the research and clinical realms exist surrounding each of these unknowns. There are three main, competing hypotheses of PTLDS pathogenesis. The first hypothesis posits the potential for an ongoing host inflammatory response independent of ongoing infection as suggested by molecular mimicry in antibiotic refractory late Lyme arthritis and anti-neuronal antibodies in PTLDS [30, 31]. Alternatively, inflammation may be driven by either occult persistent infection, as suggested by a recent mouse model of antibiotic-treated *Borrelia burgdorferi* infection [32]. These biologic explanations warrant further research that falls outside the scope of this paper.

Drawing from the ubiquity of patient-reported symptoms in the general population, a second set of hypotheses posit that these symptoms do not represent an elevation in the expected base rate and may be falsely attributed to Lyme disease exposure. While two retrospective cohort studies offer conflicting results [18, 33], a more recent meta-analysis found a higher prevalence of symptoms among patients with a history of Lyme disease compared with controls [34]. Although direct comparison is limited by methodological variability, our rates of self-reported fatigue (36%) and sleep disruption (23%) were higher than those reported for incidence in a general medical population (3% fatigue, 1% sleep disruption) [35]. Broadly defined widespread pain was reported by 20% of our cohort at 6 months, in contrast to a 1% incidence of diagnosed fibromyalgia in the general population [36]. To further compare prevalence of PTLDS symptoms to the population base rate, a sample of matched controls from the same underlying population is needed.

Finally, the psychological hypothesis is based on the premise that individuals with PTLDS may have been more vulnerable as a result of either poor adjustment or coping to having Lyme disease, or by pre-existing psychological disorders, which was born from the literature suggesting that a history of psychological trauma may pre-dispose individuals to develop “medically unexplained symptoms” [37]. This expanded to including the presence of clinical depression based on cross-sectional studies that have found that individuals with PTLDS experience mood symptoms [38–40]. However, not all studies have found that the level of depressive symptoms meets criteria for clinical depression [38] or that the depressive symptoms are related to other PTLDS symptoms [40, 41].

In our cohort, a relatively low proportion (less than 10%) of individuals reported symptoms of depression upon interview across the study period, and initial BDI-II scores did not differ statistically by later PTLDS status. Although these differences were significant after 6 months, mean scores for both groups at 6 months remained at the low end of the “minimal depression” range, only two participants had scores higher than this cutoff, and the majority of symptoms endorsed were somatic. This low rate of depression was likely influenced by our exclusion of pre-existing depression; however, it may also indicate that depressive symptomatology does not play a marked role in PTLDS during the first 6 months. These findings agree both with community-based studies that have failed to find significant elevations in depression scores among patients with a history of Lyme disease [18] and the aforementioned study of post-infectious syndromes [28] that failed to find an association with depressive symptomatology.

### Limitations of the current study

Although our high retention rate allowed for follow-up of ≥95% of the sample at each time point, our study remains limited by the small sample size of our cohort overall. In addition, this study only focused on the most characteristic and easily diagnosed manifestation of early Lyme disease, an EM rash. Patients with other presentations were excluded, as were patients with comorbid conditions that can produce symptoms similar to those found in PTLDS. The latter criteria allowed us to track the development of new symptoms over time among a relatively healthy cohort; however, it may also limit generalizability to community practice where many individuals have complex comorbid histories, including pre-existing depression. Despite this limitation, we feel that our findings are generalizable to the group of previously healthy individuals who represent the highly active individuals at highest risk of tick bites from their outdoor activities and lifestyles. Lastly, a predictive relationship between the triggering event of infection with Lyme disease and the onset of persistent symptoms cannot be established. Overall, future prospective studies including matched control groups and a diversity of comorbidities will allow for more detailed analysis and are needed to confirm our findings.

Similar to other post-infectious syndromes, the current literature reveals many unknowns surrounding PTLDS. We suggest that the present study lays the groundwork for a better understanding of signs, symptoms, and outcomes, and propose that future research take an integrative approach to examining PTLDS disease presentation, symptomatology, and impact on life functioning. Finally, we hope that viewing PTLDS as the result of multiple mechanisms will inform the field’s investigation of this syndrome and the design of appropriate interventions.

## Abbreviations

PTLDS: Post-treatment Lyme disease syndrome
EM: Erythema migrans
CBC: Complete blood counts
CMP: Complete metabolic panel
BDI-II: Beck Depression Inventory, Second Edition
